# Interaction of Insecticides and Fungicides in Bees

**DOI:** 10.3389/finsc.2021.808335

**Published:** 2022-01-25

**Authors:** Antonia Schuhmann, Anna Paulina Schmid, Sarah Manzer, Janna Schulte, Ricarda Scheiner

**Affiliations:** ^1^Behavioral Physiology and Sociobiology, University of Würzburg, Würzburg, Germany; ^2^Institute of Biology and Environmental Sciences, University of Oldenburg, Oldenburg, Germany

**Keywords:** insecticide, fungicide, honeybee, wild bee, interaction, synergistic effects, neonicotinoid

## Abstract

Honeybees and wild bees are among the most important pollinators of both wild and cultivated landscapes. In recent years, however, a significant decline in these pollinators has been recorded. This decrease can have many causes including the heavy use of biocidal plant protection products in agriculture. The most frequent residues in bee products originate from fungicides, while neonicotinoids and, to a lesser extent, pyrethroids are among the most popular insecticides detected in bee products. There is abundant evidence of toxic side effects on honeybees and wild bees produced by neonicotinoids, but only few studies have investigated side effects of fungicides, because they are generally regarded as not being harmful for bees. In the field, a variety of substances are taken up by bees including mixtures of insecticides and fungicides, and their combinations can be lethal for these pollinators, depending on the specific group of insecticide or fungicide. This review discusses the different combinations of major insecticide and fungicide classes and their effects on honeybees and wild bees. Fungicides inhibiting the sterol biosynthesis pathway can strongly increase the toxicity of neonicotinoids and pyrethroids. Other fungicides, in contrast, do not appear to enhance toxicity when combined with neonicotinoid or pyrethroid insecticides. But the knowledge on possible interactions of fungicides not inhibiting the sterol biosynthesis pathway and insecticides is poor, particularly in wild bees, emphasizing the need for further studies on possible effects of insecticide-fungicide interactions in bees.

## Introduction

Insect pollination is one of the most essential ecosystem services, with more than 75% of all crops being pollinated by insects (1, 2). Honeybees and wild bees, in particular, are indispensable pollinators of agricultural crops and natural ecosystems worldwide (3–5). The economic value of insect pollination in Europe and the United States alone is estimated at several hundred billion Dollars per year (6). In addition to climate change, habitat loss due to agricultural intensification and invasive species, the major factor in their decline is the intensive use of agrochemical plant protection products (PPPs) (5, 7). Within the group of PPPs, there are several subgroups such as fungicides, rodenticides, herbicides or insecticides. All of these products are biocidal formulations used to protect plants from pests, weeds and other diseases (8). The looming pollination crisis (9) has stimulated a general debate on the safety of PPPs and intense studies on unwanted side effects of agrochemicals on beneficial insects.

Currently, more than 1,100 PPPs (mostly fungicides, insecticides and herbicides) are marketed in European countries alone (10). They pose a continuous damage to ecosystems, because many PPPs applied do not reach the target species (11) or accidentally target beneficial insects like pollinators or even aquatic invertebrates (12). While analysis of PPP residues in wild bees is in its infancy, honeybee colonies provide many opportunities to detect plant protection residues in stored pollen (“beebread”) or honey. Residues of agrochemicals are frequent in pollen and honey stores. In the annual German bee monitoring analysis of 2017-2019^1^ (13), for example, 96.1% of the 129 beebread samples contained PPPs. In these samples, 90 different PPPs were detected, with fungicides, herbicides and insecticides being registered in almost every second sample. Worldwide, more than 160 different PPPs have been detected in honeybee colonies (7). Again, insecticides and fungicides were the dominant PPPs (14). This demonstrates that bees are chronically exposed to a cocktail of PPPs both during larval development and as adults (14, 15).

A major route of exposure is through oral contact with contaminated pollen, nectar and guttation droplets produced by plants. Importantly, the PPPs are not only present in flowers in high concentrations directly after spraying but also after systemic treatments, for example when applied as seed coating (16, 17). The PPPs are absorbed by the plant *via* the xylem and are transported through plant tissues into pollen, nectar and guttation droplets (18). Because most PPPs are highly persistent in soil and soil water, they can accumulate in pollen and nectar through this path, such as has been demonstrated for wildflowers near crops (14, 19). In addition, PPP residues can accumulate in irrigation waters, rivers, wetlands and puddles (20). Honeybees and wild bees drink from the contaminated water, thereby taking the PPPs in (8).

Honeybees and some wild bees forage over long distances (21, 22) or in mixed landscapes, encountering numerous different PPPs even within one foraging bout. But they can also encounter a cocktail of PPPs within the same flower (23) or in one puddle they drink from (20). Both honeybees and wild bees can accumulate dozens of different PPPs in varying concentrations, depending on the landscape composition in the environment of the nest. These cocktails are either taken up directly by the foragers or are fed to the brood or hive mates (14, 24). Despite the high probability of exposure to a mixture of pesticides, honeybees and wild bees have hardly been tested for unwanted effects of PPP combinations. The combination of insecticides and fungicides is a very realistic scenario since both are frequently applied onto the same crops sequentially or in a tank mixture.

In addition, there are other knowledge gaps on side effects of PPPs on non-target organisms. The majority of studies investigate PPP action under artificial conditions in the laboratory but not in the field. The focus of the studies is on the honeybee and we lack details on PPP action on wild bees. Most investigations study lethal doses and mortality, while sublethal effects are insufficiently studied. Closing these knowledge gaps is urgent, because these factors most likely have a large share in the decline of bees and other pollinators (25–28).

The comparison of effects on honeybees and wild bees is not trivial. There are several differences between honeybees and some wild bees that need to be taken into consideration. For example, wild bees frequently show a different activity window compared to honeybees, both during the day and throughout the year (29), leading to different PPP uptakes. Most wild bees have a smaller body size than honeybees (30). A dose that is not toxic for honeybees might still induce severe effects in wild bees. The life cycle of some wild bees can also lead to dose-dependent differences. Most wild bees are solitary insects—in contrast to honeybees. While in a honeybee hive conspecifics might compensate for possible side effects of PPPs, this is impossible in solitary wild bees (30). This illustrates that the comparison between honeybees and solitary wild bees is difficult. However, the comparison between honeybees and bumblebees is easier to make, and bumblebees are among the predominant pollinating wild bees in agriculture, especially in greenhouse crops (31). Both bees display a social lifestyle and have a comparable body size (30, 32).

## Insecticides

### Neonicotinoids

**Figure 1:**
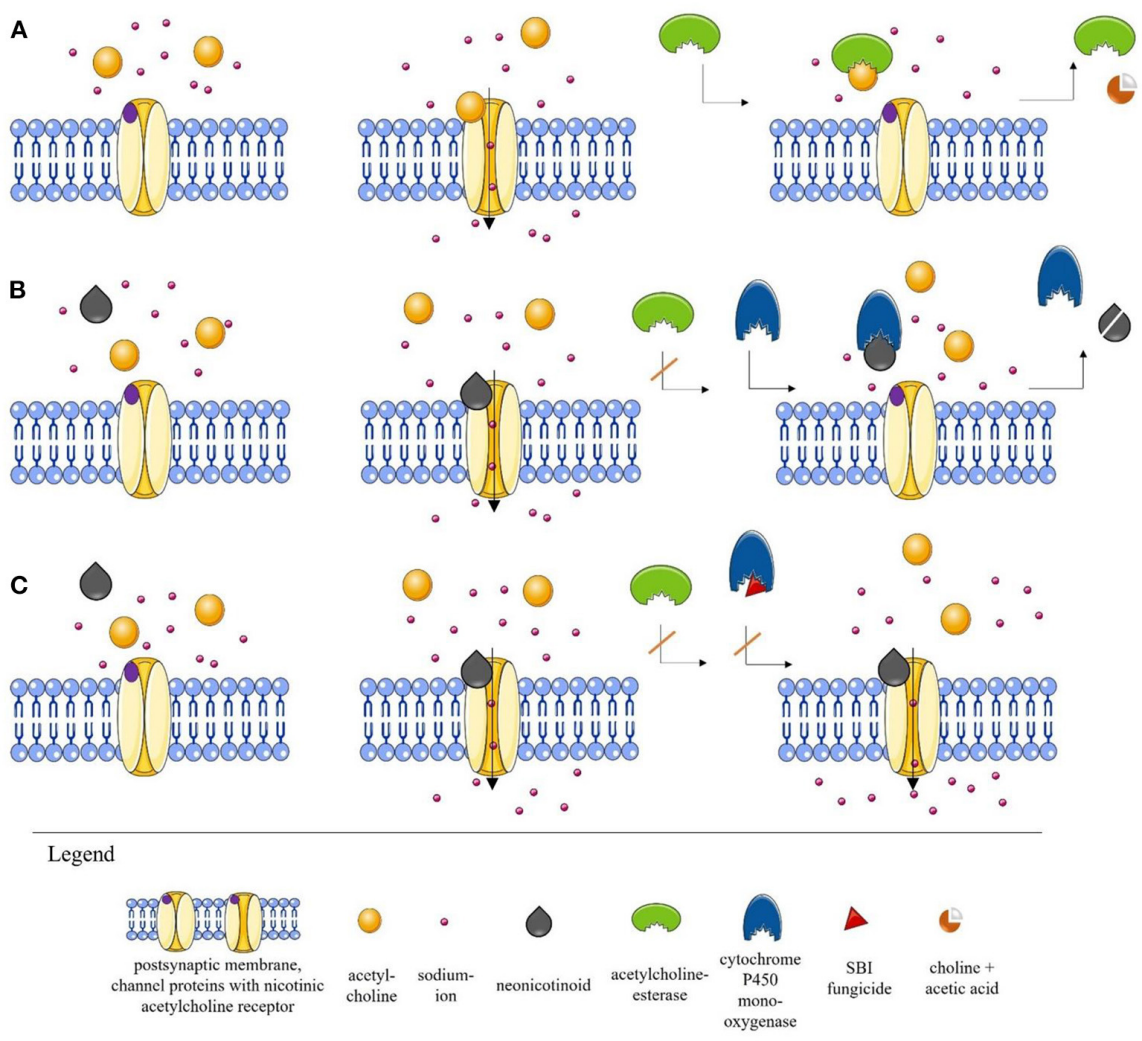
Simplified illustration of the process at the nicotinic acetylcholine receptor (nAChR) **(A)**. Normal scenario at the nAChR. When acetylcholine (ACh) is released into the synaptic cleft, it binds to the postsynaptic nAChR, thereby initiating the opening of the channel and the induction of a sodium influx, which ensures stimulus transmission. ACh is then cleaved by acetylcholinesterase (AChE) into choline and acetic acid and removed from the synaptic cleft (35, 38, 42, 47). **(B)** Scenario in the presence of a neonicotinoid. Neonicotinoids are agonists of postsynaptic nAChRs. The neonicotinoid blocks the receptor, preventing ACh accumulation. Since the AChE cannot bind to the neonicotinoid, the channel remains open and stimulus transmission is interrupted. In the optimal case, however, detoxification enzymes such as cytochrome P450 monooxygenase (P450) are able to degrade the toxic substances (36–42). (The detoxification mechanism is a complex process that has been simplified here by using only P450 for illustration purposes.) **(C)** Scenario in the presence of a neonicotinoid and a sterol biosynthesis inhibitor (SBI) fungicide. After the blockade of the receptor by the neonicotinoid, the channel remains open because the fungicide inhibits the detoxification enzyme P450 by modifying the active center. The permanent opening can lead to serious effects for insects. The direct effect of a fungicide on an insect is still largely unknown. However, such interactions that can lead to synergistic effects have already been described (39, 44–46). The illustrations were partly built using Servier Medical Art images [Servier Medical Art by Servier (https://smart.servier.com/)^2^. Servier Medical Art by Servier is licensed under a Creative Commons Attribution 3.0 Unported License] (43).

The insecticides which have attracted most attention with respect to adverse side effects on beneficial insects are the neonicotinoids (14, 24, 33, 34). They are structurally similar to nicotine and target postsynaptic excitatory nicotinic acetylcholine receptors (nAChRs) of insects, causing paralysis due to overstimulation of neurons (Figures 1A,B) (7, 36, 47, 48). When bees consume neonicotinoids, they can have severe problems in motor behaviors (49), in orientation and flight performance (34, 50–53) and display severe learning deficits (54–56), among other adverse effects. It is astonishing that neonicotinoids are still considered as relatively safe for non-target organisms, given that numerous negative effects have been reported for a variety of organisms including birds and mammals. The neonicotinoid acetamiprid, for example, was shown to induce a cytotoxic effect on mammalian cells (57). Imidacloprid, thiamethoxam, and clothianidin led to reduced food intake and associated weight loss in eared doves and it was shown that the increased use of neonicotinoids in general reduced bird biodiversity (58, 59). The neonicotinoids imidacloprid and thiamethoxam can even lead to cyto- and genotoxic effects in plants (60). Neonicotinoids are not a uniform chemical group. Structurally, they can be distinguished in two types: nitroguanidine and cyanoamidine neonicotinoids. Those of the first group contain N-nitro-groups in their structure, which contain oxygen atoms, making them more polar and reactive. Imidacloprid, clothianidin and thiamethoxam belong to this group. They are generally more toxic to bees than neonicotinoids of the second group, which comprise acetamiprid and thiacloprid. These contain cyanoamidine groups in their particles instead of the nitro group. Since the cyanoamidine group does not include oxygen atoms, they are less reactive and therefore less toxic (44, 61).

While the first three neonicotinoids were restricted in use in 2013 (62)^3^ and completely banned from field use in the EU in 2018 (63–65)^4^^−^^6^, the less toxic cyanoamidine neonicotinoids were in use up to recently. But compelling evidence of aversive effects of thiacloprid (51, 55, 66) has meanwhile led to a European ban of this insecticide in greenhouse and field (67)^7^. Nevertheless, in 2020, the neonicotinoids imidacloprid, clothianidin, thiamethoxam and thiacloprid received emergency authorisations for sugarbeet cultivation in several European countries (68)^8^. Such emergency authorisations can lead to persistent residues in the field. In Germany, thiacloprid has been detected in beebread in 30% of samples analyzed in 2020 (69) (see text footnote 1). Thiamethoxam and clothianidin have been detected in pollen even more frequently (86.7%) and in almost all nectar samples (98.6%)—although mostly at very low concentrations (70). Acetamiprid and thiacloprid have been detected in 20% of beebread samples in Poland (71). However, a significant proportion of the food consumed worldwide is grown in Asia and the Americas (72)^9^. In these places, PPPs like neonicotinoids can still be detected frequently in alarming concentrations. In the US, clothianidin has been detected in all agricultural areas studied and imidacloprid has been found in almost every urban water (73). More detailed studies in New York State and California showed that contamination of aquatic systems with neonicotinoids continues to increase (74). Studies in Northern Belize found neonicotinoids in 68% of soil samples and 47% of sediment samples (75). In China, neonicotinoids like imidacloprid and thiamethoxam have been detected in river systems (76) and 40% of honey samples studied contained at least one neonicotinoid (77). Imidacloprid has also been detected in Japan in harmful concentrations for aquatic invertebrates (78). In fact, 86% of all samples tested in North America and 80% of samples in Asia were contaminated with neonicotinoids in a recent investigation by Mitchell et al. (24).

Some PPPs containing the neonicotinoid acetamiprid must no longer be sprayed into open rape flowers, at least in some European countries [e.g., Germany (79)]^10^ due to its toxicity for bees (80, 81). However, acetamiprid can still be used as systemic PPP, leading to residues in pollen, nectar and guttation droplets. In addition, some residues will still reach open rapeseed flowers even though the neonicotinoid should only be sprayed on plants prior to full bloom, because a rapeseed field does not bloom evenly.

In addition to these exceptions to the ban of neonicotinoids, novel substitutes for neonicotinoids such as flupyradifurone or sulfoxaflor have reached the market (82–85). These bind to the same neurotransmitter receptors in the brain of insects and even though they generally appear to be less toxic to honeybees, in Germany they can only be used in greenhouses to protect honeybees and wild bees (86)^11^. Evidence is accumulating that these substances, too, can have adverse effects on bees (82–84, 87, 88).

### Pyrethroids

Pyrethroids are the second most important insecticide class. Synthetic pyrethroids are derived from one of six natural pyrethrins, i.e., cinerin I, of the pyrethrum flower, *Tanacetum cinerariifolium* (89). They act on voltage-gated sodium channels in the membranes of nervous cells (37, 90, 91), leading to a permanent depolarization of the axon membrane. Application of pyrethroids leads to hyperexcitatory symptoms (92), paralysis and ultimately to a knockout (93, 94). Since pyrethroids are rapidly metabolized in insects by the cytochrome P450 detoxification pathway, they are generally considered not to pose a high risk for bees (37). On the other hand, they have relatively higher LD50 values for insects compared to neonicotinoids and share similar sub-lethal effects on bees (95, 96). Typical pyrethroid insecticides include tau-fluvalinate, cyfluthrin, allethrin, permethrin, deltamethrin, lamda-cyhalothrin, and alpha-cypermethrin.

Since the ban of most neonicotinoids from field use, many farmers have switched to pyrethroids along with alternative pest control methods (97). In Germany, tau-fluvalinate can be considered as the second most frequent insecticide in beebread, followed by deltamethrin (69) (see text footnote 1). In recent years, lamda-cyhalothrin has been used on 43% of oilseed rape fields in the UK (98). In France, tau-fluvalinate belonged to the substances that were found most frequently in beeswax (61.9% of all samples tested). The pyrethroid cypermethrin has been detected in 21.9% of samples, while deltamethrin appears to be used less frequently (2.4% of samples) (99). In North America, Thailand and Taiwan the pyrethroid fluvalinate was detected in concentrations of 2,670 μg/kg (15), 47 μg/kg (100) and 16,260 μg/kg (101) bee pollen respectively. Permethrin (70 μg/kg) was found in bee pollen in Brazil (102) and lamda-cyhalothrin was detected in Chinese bee pollen in high concentrations (12,476 μg/kg) (103).

## Fungicides

Fungal diseases are the greatest threat to crops worldwide. Thus, it is not surprising that the use of fungicides has been increasing constantly over the last decades (104–106). They are often applied during bloom (107), so that bees have direct contact with them during foraging (69) (see text footnote 1). But fungicides are also among the most common agrochemical contaminants found in beeswax and pollen reserves of honeybee hives, indicating that colony exposure likely extends beyond the bloom period (15). Of the large number of fungicides, boscalid is one of the most frequently detected fungicides in bee products in Germany and Poland (69, 71) (see text footnote 1). Other frequent fungicides in beebread studied from German honeybee colonies are azoxystrobin, tebuconazole, prothioconazole and dimoxystrobin. The same trend can be observed in the United States with boscalid and azoxystrobin residues being detected frequently (108) and in other European countries showing similar frequencies of these two fungicides (109). Furthermore, azoxystrobin was detected in Uruguay (5.5 μg/kg bee pollen) and propiconazole was found in the USA (17 μg/kg bee pollen) (110, 111).

Fungicides are generally considered harmless for bees based on short-term toxicity tests (112) and the fact that oral and contact LD50s for fungicides measured in individual bees are usually at least four orders of magnitude greater than concentrations found in honeybee food stores (113). However, standard toxicity tests often disregard sublethal effects (114). Sublethal fungicide effects, however, may cause severe stress to bees (115). Fungicide exposure, for example, can lead to negative effects on food consumption, immune response and metabolism of insect pollinators (116). A recent study by DesJardins et al. (117) demonstrates that the fungicide Pristine® with the active ingredients boscalid (25%) and pyraclostrobin (13%) can have a severe impact on honeybee cognition. The same PPP reduced life span and led to an earlier onset of foraging in another study (118). These examples suggest that fungicides can have similar sublethal effects on bees as neonicotinoids, although their mode of action is completely different and they are still considered as harmless for insects. In addition, fungicides might exert even further effects on bees. For example, some bee species live in a mutualistic relationship with a fungus that is essential for the development of their larvae. Fungicides could damage their essential fungus (119). Furthermore, it is known that some fungicides such as boscalid interfere with the respiratory chain of fungi which results in a disruption (38, 120). Thus, another possibility of how fungicides could affect pollinators is that they disrupt the respiratory chain in insects. This could result in insufficient energy provisioning for various activities, resulting in sublethal effects. Moreover, fungicides can influence the microbiome of some organisms like mice or soil animals (121–123). Presumably, fungicides can also affect the microbiome of honeybees, thereby influencing their immune system.

These findings underpin the urgent need for more controlled studies on the effects of fungicides on pollinators.

## Combinations of Neonicotinoids With Sterol Biosynthesis Inhibiting Fungicides

While we still lack information about possible side effects of the majority of fungicides on honeybees and wild bees, evidence is accumulating that some fungicides can have special additive or synergistic effects on bees when co-applied with neonicotinoids (124, 125). An additive effect occurs when the cumulative effect of a fungicide and of an insecticide equals the sum of the individual effects of each substance, while a synergistic effect indicates a significantly larger effect than that of the sum of individual effects (124, 125). Crops are usually treated frequently against diverse pests, so that there is a good chance of fungicides and insecticides being applied together in a tank mixture or with only a short time interval in between (23, 126). One critical combination is the mixture of neonicotinoids or pyrethroids with azole fungicides, because the latter inhibit the sterol biosynthesis pathway (“sterol biosynthesis inhibiting fungicides,” hereafter: SBI fungicides) (127, 128). SBI fungicides inhibit the cytochrome P450 mediated ergosterol synthesis, which is essential for cell membrane functioning in fungi (39, 44). However, they are also capable of inhibiting the enzyme cytochrome P450 monooxygenase (P450) of honeybees and wild bees, thereby disrupting their detoxification pathway (45, 46). Since cytochrome P450 enzymes in bees are responsible for both the detoxification of naturally occurring phytochemicals (129) and the detoxification of different insecticides (130), the combination of SBI fungicides with some insecticides can trigger synergistic effects and thus increase their toxicity to bees (Figure 1C) (40, 44). Although bees also have other metabolic pathways, many toxins are metabolized *via* the cytochrome P450 degradation pathway.

Intriguingly, the probability for synergistic toxic effects of a neonicotinoid-SBI fungicide combination appears to depend on the class of neonicotinoids. Joint application of the cyanoamidine neonicotinoids acetamiprid or thiacloprid, which themselves have a relatively low toxicity for bees, with the SBI fungicides triflumizole or propiconazole increased the acute contact toxicity in bees several hundred-fold. A combination of thiacloprid and triflumizole even led to a 1,141-fold increase in toxicity (44)! Similar results were demonstrated for a combined application of acetamiprid and the SBI fungicide propiconazole (131). Chronic exposure to these two substances caused synergistic negative effects on the mortality and weight of Asian honeybees (*Apis cerana*) (17).

Imidacloprid, which belongs to the nitroguanidine neonicotinoids, in contrast, only showed a weak increase in toxicity when combined with the SBI fungicides triflumizole or propiconazole (44), although it belongs to the group of neonicotinoids which themselves are frequently more toxic to bees than the cyanoamidine neonicotinoids. Another study by Yao et al. (132) showed a similar result when co-applying the nitroguanidine neonicotinoid clothianidin jointly with the SBI fungicide tetraconazole.

Similar to what has been reported for honeybees, combinations of cyanoamidine neonicotinoids with SBI fungicides led to stronger or synergistic effects on wild bees compared to combinations of nitroguanidine neonicotinoids. The cyanoamidine neonicotinoid acetamiprid and the SBI fungicide fenbuconazole, for example, showed significantly negative synergistic effects on the acute toxicity of the horned-face bee *Osmia cornifrons*, while the effects of the nitroguanidine neonicotinoid imidacloprid in combination with the SBI fungicide only led to slight effects (133). Co-application of imidacloprid and the SBI fungicide imazalil to bumblebee (*Bombus terrestris*) workers also did not reveal any effect on acute oral toxicity or feeding rate (127). However, there are some exceptions. Studies exposing *Bombus terrestris* workers to a mixture of the nitroguanidine neonicotinoid thiamethoxam and the SBI fungicide imazalil showed a significant synergistic effect on mortality but no effect on feeding (127) and joint application of thiamethoxam and the SBI fungicide myclobutanil increased acute toxicity in *Bombus impatiens* bees by the factor 2.38 (128). The nitroguanidine neonicotinoid clothianidin induced a significantly higher mortality not only in honeybees, but also in bumblebees (*Bombus terrestris*) and solitary bees (*Osmia bicornis*) when co-applied orally with the SBI fungicide propiconazole. Furthermore, the mixture led to slow ovary maturation and decreased longevity in *Osmia bicornis* (134, 135).

Some of the novel substitutes for neonicotinoids have also been tested for additive or synergistic effects in combination with SBI fungicides. Although the novel substances such as flupyradifurone (marketed under the name of Sivanto®) belong to different chemical classes as the classical neonicotinoids (butenolides in the case of flupyradifurone), they bind to the same nicotinic acetylcholine receptors in the nervous system of insects (136). Not surprisingly, a combined application of the SBI fungicide propiconazole and flupyradifurone synergistically increased the acute mortality of bees and led to acute synergistic sublethal effects such as abnormal coordination, hyperactivity and apathy (87).

## Combinations of Pyrethroids and SBI Fungicides

Pyrethroid insecticides rely on the same enzyme for detoxification processes as neonicotinoids. It is therefore not surprising that these PPPs also show an enhanced toxicity when applied in combination with SBI fungicides. Further, pyrethroids are highly hydrophobic. Their toxicity is therefore often larger by contact than by oral exposure (14). Honeybees sprayed with sublethal doses of the pyrethroid deltamethrin and the SBI fungicide prochloraz, for example, displayed a synergistically enhanced acute mortality, while either compound on its own did not show any effects on mortality (45, 137). The combination of the same two compounds or of deltamethrin and the SBI fungicide difenoconazole caused an acute synergistic hypothermia in an experiment investigating thermoregulation (138). Studies on semi-isolated hearts of honeybees further showed a synergistic cardiotoxic effect of deltamethrin in combination with prochloraz (139). The pyrethroid lambda-cyhalothrin similarly showed synergistic effects on the acute mortality of honeybees in combination with the SBI fungicides prochloraz, propiconazole, imazalil and others (41, 46). Of these, the strongest synergistic effect was found in combination with propiconazole, with a synergistic ratio of 16.2. Furthermore, lamda-cyhalothrin showed an increased acute toxicity when applied to honeybees in combination with the SBI fungicides flusilazole, difenoconazole, tebuconazole, prochloraz and propiconazole. The strongest effect was detected in combination with prochloraz. Furthermore, the oral combinations of the pyrethroid alpha-cypermethrin and the SBI fungicides prochloraz and propiconazole led to an increase in acute toxicity, with the most pronounced effects induced by prochloraz (126). The difference in the study design was that Pilling and Jepson (46) applied the PPPs via contact, while Thompson and Wilkins (126) used oral exposure. Wernecke et al. (140) showed synergistic toxic effects with mortality rates up to 100% when they applied lambda-cyhalothrin in combination with the SBI fungicide tebuconazole. The pyrethroid cypermethrin showed a significant synergistic effect in combination with the SBI fungicide imazalil in acute mortality tests using the bumblebee *Bombus terrestris*. The foraging rate, however, was unaffected (127). A combination of the pyrethroid bifenthrin with the SBI fungicide difenoconazole slightly enhanced mortality in bumblebees, while a combination with the SBI fungicide myclobutanil enhanced mortality by the synergy ratio of 11 (128).

The above studies and others have likely contributed to political decisions in the EU, which strongly restrict the use of neonicotinoid insecticides (62, 67) (see text footnote 3,7), thereby leading to the expiration of approval of most neonicotinoids by the end of 2020. The only exception is acetamiprid. To the best of our knowledge, Germany is the only country within the EU which went as far as prohibiting the combination of neonicotinoids and SBI fungicides and of pyrethroids and SBI fungicides on plants that are visited by bees (141–144)^12^^−^^15^. The combination of pyrethroids, the neonicotinoid acetamiprid or of new substitute products for neonicotinoids and SBI fungicides is therefore still likely to occur in many countries within the EU and outside, putting honeybees and wild bees at risk.

## Combinations of Neonicotinoids and Non-SBI Fungicides

Diverse insecticides and fungicides have frequently been detected in beebread, honey and wax (14, 145). Even though many fungicides and insecticides may not be sprayed together in a tank mixture, at least in some countries, they can be applied sequentially, so that bees will still consume them together while collecting pollen or nectar. Sometimes, the neonicotinoid may be present in guttation drops after systemic application while the fungicide has been sprayed on the flowers, so that a bee collecting both water and pollen might come into contact with both substances within one foraging bout (23, 126). Therefore, the question remains whether a combined application of neonicotinoids and non-SBI fungicides has any negative effects on honeybees and wild bees. Studies on these combinations and their effects on pollinators are relatively new and still rare.

**Figure 2:**
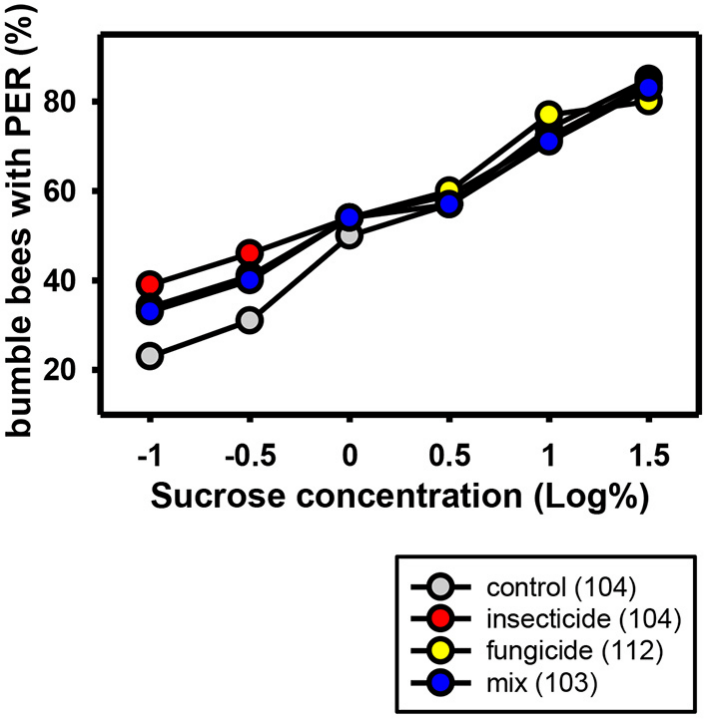
Responses to increasing concentrations of sucrose of commercial bumblebees (*Bombus terrestris*) chronically treated with the neonicotinoid Mospilan® [active ingredient: acetamiprid (2,000 ppb)], the non-SBI fungicide Cantus® Gold [active ingredients: boscalid/dimoxystrobin (50/50%) (150 ppb boscalid/150 ppb dimoxystrobin)], a mixture of both PPPs or a control solution containing only sucrose and water. The bumblebees were anesthetized on ice and harnessed in holders. After an interval for adaptation, the antennae of the bumblebees were stimulated with several sucrose concentrations in ascending order. It was noted if individual bumblebees showed a proboscis extension response (PER) to the sucrose stimulus. The number of bumblebees tested is shown in brackets for each treatment. The percentage of bumblebees extending their probosces to the different sugar concentrations increases with sugar concentration. Field realistic concentrations of the non-SBI fungicide, the neonicotinoid or a combination of these had no effect on sugar responsiveness.

One study of the neonicotinoid thiamethoxam in combination with the non-SBI fungicide carbendazol showed an effect on sugar responsiveness and orientation behavior of honeybees. However, thiamethoxam showed this effect in the solo application too, thus it cannot be concluded that there is a synergistic effect of the interaction between thiamethoxam and carbendazol and the effects on orientation and sugar responsiveness was recovered in all bees 1 week after exposure to the PPPs (52). The same applies to the combination of the neonicotinoid thiamethoxam and the non-SBI fungicide picoxystrobin. They show a chronic toxicity effect on newly emerged honeybees and an overload of the hepato-nephrocitic system when applied in combination (146). Again, the two toxins showed this effect in the solo application, too. Schmuck et al. (40) showed that the neonicotinoid thiacloprid displayed no negative synergistic effects when applied jointly with non-SBI fungicides (tolylfluanid, mancozeb, azoxystrobin). The only exception was cyprodinil with a small additive effect. Wernecke et al. (140) showed that tank mixtures containing the non-SBI fungicides boscalid and dimoxystrobin and the neonicotinoid thiacloprid had no effect on bee toxicity both in laboratory, semi-field and field standard assays. Likewise Manning et al. (131) showed that the non-SBI fungicides pyraclostrobin and boscalid did not enhance toxicity of the neonicotinoid acetamiprid in honeybees. However, a study of Tsvetkov et al. (147) showed that the acute toxicity of the neonicotinoids clothianidin and thiamethoxam was increased when they were co-applied orally in combination with field realistic concentrations of the non-SBI fungicide boscalid. The LD50 was significantly reduced and both insecticides became nearly twice as toxic. Studies on the effects of a combined application of neonicotinoids and non-SBI fungicides in wild bees are not available. Our own experiments analyzing the sucrose responsiveness of bumblebees after chronic oral exposure to the neonicotinoid acetamiprid and the non-SBI fungicides boscalid and dimoxystrobin in sublethal concentrations did not reveal any negative effects (Figure 2).

These experiments jointly show that the combination of neonicotinoids and non-SBI fungicides appears not to increase toxicity of the neonicotinoids. They support the notion that the enhanced toxicity of neonicotinoids in combination with SBI fungicides relies mainly on the inhibition of P450 enzymes and not on additional factors.

## Combinations of Pyrethroids and Non-SBI Fungicides

Only very few studies have been published on the combined effects of pyrethroids and non-SBI fungicides. The non-SBI fungicides carbendazol and iprodione+thiophanate-methyl did not increase the toxicity of the pyrethroid insecticides alpha-cypermethrin and lamba-cyhalothrin in honeybees, while chlorothalonil was the only non-SBI fungicide that showed an effect on the toxicity of alpha-cypermethrin and lambda-cyhalothrin (126). As chlorothalonil is metabolized in mammals *via* the enzyme P450 (37), it stands to reason that the enzyme might also play a role in the detoxification of the fungicide in honeybees. Thus, the increased toxicity could be due to the competition for P450 between chlorothalonil and the pyrethroids.

## Discussion

**Figure 3:**
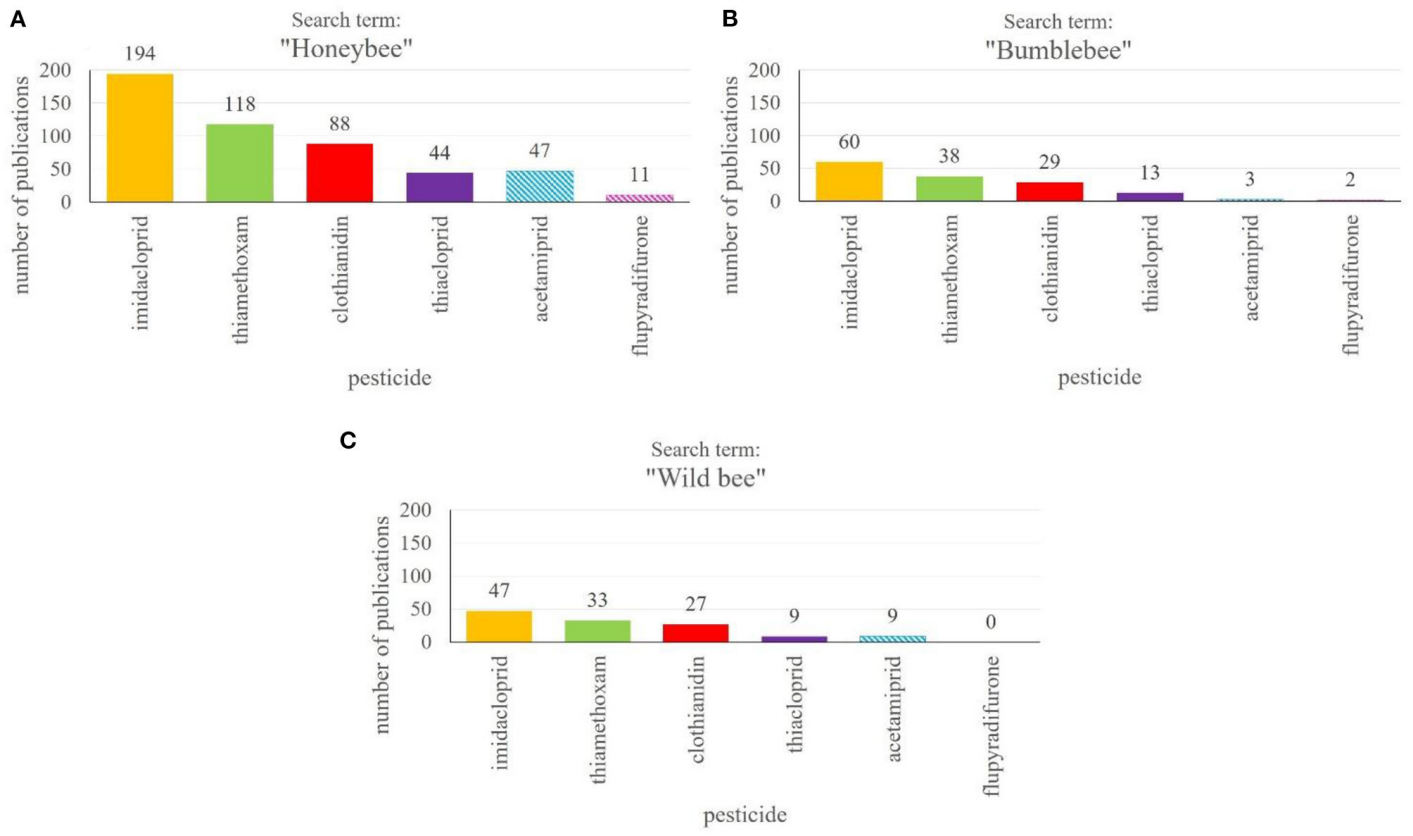
Number of publications on different neonicotinoid insecticides and novel products binding to nicotinic acetylcholine receptors. The search for publications was performed in the Web of Science Core Collection^18^ using the “name of the pesticide” and “honeybee” **(A)**, “bumblebee” **(B)** or “wild bee” **(C)**. The absolute numbers of publications are given above the columns. Fully filled columns show already banned pesticides, while striped columns represent currently approved PPPs.

Honeybees and wild bees, along with other pollinators, depend on protection in agricultural landscapes due to heavy use of plant protection products, posing enourmous stress on bees worldwide (2, 7, 148). Policy makers, on the other hand, rely on independent scientific research on possible negative side effects of PPPs for a wide range of insects and in combination with other PPPs, because the agrochemicals, for which there is a high demand in food production industry, are naturally only tested for adverse side effects on beneficial insects to a small degree during the approval process and sublethal effects are hardly studied (25, 149, 150). Interestingly, there is a strong positive correlation between the number of papers published on a certain PPP and the likelyhood that it will be banned from field use (Figures 3A–C). At least, this is highly apparent for the main class of insecticides, the neonicotinoids. According to our literature search in the Web of Science Core Collection (151), the largest number of publications focusses on the three neonicotinoids imidacloprid, thiamethoxam and clothianidin (Figures 3A–C). They are followed by studies investigating side effects of thiacloprid and acetamiprid, but the latter two have been studied to a considerably lower extend. While imidacloprid, thiamethoxam and clothianidin are meanwhile largely banned from use in the EU (63–65) (see text footnote 4-6) the approval of acetamiprid was renewed until 2033 (152)^16^. In Germany, however, there is the restriction that some PPPs containing acetamiprid may only be sprayed on plants just prior to full bloom (79)^17^. Thiacloprid was banned from outdoor and greenhouse use recently (67).

We can therefore expect that residues will increase for the remaining neonicotinoids such as acetamiprid (17) and novel insecticides such as flupyradifurone (84), sulfoxaflor (85, 153), and cyantraniliprole (154). Some of them also bind to nicotinic acetylcholine receptors like the neonicotinoids [flupyradifurone: (136, 155)] [sulfoxaflor: (153)] or modulate the ryanodine receptor [cyantraniliprole: (156)].

The studies of side effects of neonicotinoids on honeybees are comparatively rich and diverse. In addition to testing effects on mortality, they use an ample array of behavioral tests to study in detail sublethal effects on honeybee behavior. Studies on the effects of neonicotinoids on wild bees, in contrast, are rather rare (Figures 3A–C). One reason is the lack of suitable and established test procedures such as those developed for model organisms like the honeybee (*Apis mellifera*). Wild bees are not as easily available as honeybees or other social insects at different times of the year and in adequate numbers. The bumblebee is the only “wild” bee which has gained considerable attention in PPP tests, not least because numerous assays originally developed for honeybees have been adapted for bumblebees. Nevertheless, there are some important differences between honeybees and bumblebees concerning their physiology and life cycle (157, 158). Honeybees, for example, store contamined pollen or nectar inside the hive, which results in a dilution effect. Bumblebees do not show this behavior, which could lead to higher intakes per individual (159). Solitary wild bees are even more at danger to suffer from intense use of PPPs for various reasons. Their solitary lifestyle prevents compensation of possible negative effects by conspecifics. In addition, it has been assumed that honeybee workers are able to filter toxins before feeding the brood, which has not been shown in solitary insects. Furthermore, many wild bees are smaller than honeybees or bumblebees, possibly making them more vulnerable (30, 160). Since the number of studies on PPP effects on wild bees is very small (Figure 3C), our knowledge on the action and interaction of neonicotinoids and fungicides basically relies on honeybee studies and a few studies investigating bumblebees or sometimes mason bees. Assuming that wild bees have a higher vulnerability than larger bees and taking into account the great loss of wild pollinators even in national parks, we can assume that studying only model organisms and selected bees which are commercially available is insufficient to explain the effects of individual PPPs or their combination on the majority of wild bees.

Our review illustrates that neonicotinoids, which are targeted at sucking insect pests of agricultural crops (161), are harmful to honeybees and wild bees (19, 133, 162–164). Among the different neonicotinoids, those containing a cyanoamidine group (cyanoamidine type) have a lower toxicity for honeybees and wild bees than those containing a N-nitro group (nitroguanidine type). However, in combination with SBI fungicides, which increase the toxicity of neonicotinoids through inhibition of their detoxification pathway (37), the toxicity of the two classes of neonicotinoids appears to be inverted with nitroguanidine neonicotinoids being less toxic than cyanoamidine neonicotinoids. Similarly, the increased toxicity of pyrethroid insecticides when co-applied with SBI fungicides seems to be solely related to their inhibition of the detoxification pathway, as is the case in neonicotinoids.

The non-SBI fungicides, in contrast, appear not to impose a higher toxicity to honeybees and wild bees when co-applied with neonicotinoids, although only few studies have investigated this issue. Yet, the overall results are very clear and there is no hint that those fungicides interfere with detoxification mechanisms. The very few studies analyzing pyrethroids in combination with non-SBI fungicides do not suggest any additive or synergistic effects induced by the fungicides.

It needs to be pointed out that the effects reported for individual PPPs or their combinations sometimes differ drastically between studies on the same species applying the same products. Care must be taken when trying to formulate a general statement on the toxicity of such combinations or individual PPPs. One reason is the different application method, which can have gross effects on toxicity. The nitroguanidine neonicotinoid imidacloprid in combination with tetraconazole, for example, did not evoke any negative effects in honeybees when applied orally for 14 days in one study (165). The same combination of PPPs led to synergistic toxic effects when sprayed onto individuals, leading to direct contact (166). A contact application of the nitroguanidine neonicotinoid thiamethoxam in combination with the SBI fungicide tebuconazole led to a synergistic 2.6-fold increase in toxicity for honeybees. However, when applied orally to honeybees, the synergistic toxicity was much reduced (23).

A second important point in discussing the effects of single or combined PPP application concerns the concentrations and amounts of active substances. Presumably, this discrepancy between studies leads to the largest differences reported on the same substances. It is not always straightforward to estimate a field realistic dose of PPPs or active ingredients for different bee species, because there are not reliable residues known for each species. In honeybees, regular monitoring of PPP residues in beebread (stored pollen), honey, wax and in pollen collected from individual bees allows for a detailed analysis (14, 15, 37, 167–169). For bumblebees, the situation is very different with few studies having analyzed the residues of PPP in pollen collected from individuals (170). Nevertheless, the bumblebee *Bombus terrestris* is a generalist (171), i. e. the bumblebees forage on similar plants compared to honeybees and can come into contact with the same residues, but nothing is known about natural exposition of solitary wild species such as mason bees. Wildflowers near arable fields often contain higher residue levels compared to crop plants (172). Therefore, wild bees foraging on wildflowers may even experience higher exposure levels than bees foraging in agricultural crops.

In numerous studies, lethal and (less frequently) sublethal doses are determined and used for experimental analysis of toxicity. However, only rarely are they related to realistic scenarios of exposure in the field. This is more difficult when no residues have been determined, e.g., in nests of solitary bees or in the pollen collected in the corbiculae. However, it is questionable how far determining sublethal or lethal dosages represent a realistic situation for a given species. It is similarly difficult to calculate exposure concentrations from known application rules, because a large proportion of bees are more likely to come into contact with PPPs through feeding from stored nectar or pollen than by direct contact (173). In addition, a realistic scenario should either involve a larger number of PPPs than one or two, because many bee products accumulate up to 30 different PPPs (13) (see text footnote 1), or it should estimate the total amount of PPPs an individual is exposed to, e.g., by adding the amounts of different PPPs. The varied perspectives, methods and calculations of PPP amounts across experiments make it very difficult to compare different studies. Nevertheless, our abundant knowledge on PPP effects on honeybees can serve as a good approximation for possible effects on wild bees and other pollinators, when care is taken in comparisons and conclusions.

Taken together, our survey suggests that the recent ban of most neonicotinoids from field use together with the ban on tank mixtures of neonicotinoids or pyrethroids and SBI fungicides in Germany have been decisive measures to protect honeybees and wild bees from adverse side effects of these PPPs and their combinations. Nevertheless, Germany and other European countries import a large part of their food from other countries (175) and many PPPs like neonicotinoids are exported from Europe to these countries, where their application is still possible (174, 175)^19^. This is because the application regulations and safety standards concerning the use of PPPs are not developed uniformly worldwide (175, 176). Especially developing countries, often relying on food exports, have weaker food safety regulations (177). These countries often have a great biodiversity and a large variety of bees and other pollinators (178). Our review suggests that their insect diversity is severely threatened by the combined use of neonicotinoids and SBI fungicides. Further, it should be pointed out that the PPPs such as neonicotinoids, which are still in use in those countries, can be re-imported to Europe via the import of fruit and vegetables, despite strict import regulations (175).

We have no clear evidence for negative effects of combined applications of neonicotinoids or pyrethroids and non-SBI fungicides so far, but there have been only few studies investigating these effects up to now, particularly in wild bees. Our survey highlights the importance of considering interactive effects of PPPs in risk assessment, even though it will be a big challenge to investigate lethal and sublethal effects of the thousands of potential chemical combinations to which bees are exposed in the environment. Novel computer algorithms and machine learning might help to simulate these interactions when the mechanisms of actions of individual PPPs are known and taken into consideration, ultimately protecting our most important pollinators in their environment.

## Author Contributions

AS, APS, and RS provided the first draft of the manuscript. JS contributed to experimental data. SM worked at the revisions of the manuscript. All authors agreed to be accountable for the content of the work.

## Funding

This work was supported by a grant from the Bavarian State Ministry of the Environment and Consumer Protection to RS in the research network BayÖkotox (TP 2 Honig- und Wildbienen unter Stress) and by a grant of the German Federal Environmental Foundation to AS.

## Conflict of Interest

The authors declare that the research was conducted in the absence of any commercial or financial relationships that could be construed as a potential conflict of interest.

## Publisher's Note

All claims expressed in this article are solely those of the authors and do not necessarily represent those of their affiliated organizations, or those of the publisher, the editors and the reviewers. Any product that may be evaluated in this article, or claim that may be made by its manufacturer, is not guaranteed or endorsed by the publisher.

## Footnotes

^1^https://bienenmonitoring.uni-hohenheim.de (accessed October 20, 2021).

^2^https://smart.servier.com (accessed November 25, 2021).

^3^https://eur-lex.europa.eu/eli/reg_impl/2013/485/oj (accessed October 28, 2021).

^4^https://eur-lex.europa.eu/legal-content/EN/TXT/?uri=CELEX%3A32018R0783 (accessed October 28, 2021).

^6^https://eur-lex.europa.eu/legal-content/EN/TXT/?uri=CELEX%3A32018R0785 (accessed October 28, 2021).

^7^https://eur-lex.europa.eu/eli/reg_impl/2020/23/oj (accessed October 28, 2021).

^8^https://www.efsa.europa.eu/de/news/pesticides-efsa-examine-emergency-use-neonicotinoids (accessed October 28, 2021).

^9^https://www.fao.org/faostat/en/#data/TCL (accessed November 23, 2021).

^10^https://www.bvl.bund.de/SharedDocs/Fachmeldungen/04_pflanzenschutzmittel/2021/2021_03_12_Fa_Mospilan_SG_verkuerzte_AW_Raps.html (accessed October 20, 2021).

^11^https://www.bvl.bund.de/SharedDocs/Fachmeldungen/04_pflanzenschutzmittel/2020/2020_04_09_Fa_Zulassung_Sivanto_Gewaechshaus.html (accessed August 10, 2021).

^12^https://www.lfl.bayern.de/ips/recht/205171/index.php (accessed November 1, 2020).

^15^https://apps2.bvl.bund.de/psm/jsp/DatenBlatt.jsp?kennr=005655-60 (accessed October 20, 2021).

^16^https://eur-lex.europa.eu/legal-content/EN/TXT/?uri=CELEX%3A32018R0113 (accessed: October 28, 2021).

^17^https://www.bvl.bund.de/SharedDocs/Fachmeldungen/04_pflanzenschutzmittel/2021/2021_03_12_Fa_Mospilan_SG_verkuerzte_AW_Raps.html (accessed October 20, 2021).

^18^https://www.webofscience.com/wos/woscc/basic-search (accessed November 23, 2021).

^19^https://www.fao.org/faostat/en/#data/RT (accessed November 23, 2021).

## Abbreviations

ACh: acetylcholine
nAChR: nicotinic acetylcholine receptor
P450: cytochrome P450 monooxygenase
PPP: plant protection product
SBI fungicide: sterol biosynthesis inhibiting fungicide.
